# Outbreak detection for temporal contact data

**DOI:** 10.1007/s41109-021-00360-z

**Published:** 2021-02-19

**Authors:** Martin Sterchi, Cristina Sarasua, Rolf Grütter, Abraham Bernstein

**Affiliations:** 1 Department of Informatics, University of Zurich, Binzmühlestrasse 14, 8050 Zurich, Switzerland; 2 Swiss Federal Research Institute WSL, Zürcherstrasse 111, 8903 Birmensdorf, Switzerland; 3 University of Applied Sciences and Arts Northwestern Switzerland FHNW, Riggenbachstrasse 16, 4600 Olten, Switzerland

**Keywords:** Outbreak detection, Epidemic spreading, Temporal networks, Submodular functions, Greedy optimization

## Abstract

Epidemic spreading is a widely studied process due to its importance and possibly grave consequences for society. While the classical context of epidemic spreading refers to pathogens transmitted among humans or animals, it is straightforward to apply similar ideas to the spread of information (e.g., a rumor) or the spread of computer viruses. This paper addresses the question of how to optimally select nodes for monitoring in a network of timestamped contact events between individuals. We consider three optimization objectives: the detection likelihood, the time until detection, and the population that is affected by an outbreak. The optimization approach we use is based on a simple greedy approach and has been proposed in a seminal paper focusing on information spreading and water contamination. We extend this work to the setting of disease spreading and present its application with two example networks: a timestamped network of sexual contacts and a network of animal transports between farms. We apply the optimization procedure to a large set of outbreak scenarios that we generate with a *susceptible-infectious-recovered* model. We find that simple heuristic methods that select nodes with high degree or many contacts compare well in terms of outbreak detection performance with the (greedily) optimal set of nodes. Furthermore, we observe that nodes optimized on past periods may not be optimal for outbreak detection in future periods. However, seasonal effects may help in determining which past period generalizes well to some future period. Finally, we demonstrate that the detection performance depends on the simulation settings. In general, if we force the simulator to generate larger outbreaks, the detection performance will improve, as larger outbreaks tend to occur in the more connected part of the network where the top monitoring nodes are typically located. A natural progression of this work is to analyze how a representative set of outbreak scenarios can be generated, possibly taking into account more realistic propagation models.

## Introduction

Spreading processes in networks have been extensively studied in the literature. Spreading can happen in a wide variety of contexts, including animal or human epidemic spreading (Rocha et al. 2011; Bajardi et al. 2012), computer virus spreading (Pastor-Satorras and Vespignani 2001), and misinformation spreading over social media (Budak et al. 2011). The spreading phenomena often have negative implications and it is of utmost importance to implement eradication strategies to avoid an uncontrolled result (e. g., a global pandemic or a political catastrophe). Sometimes, though, the goal is to foster spreading, for example in the case of viral marketing (Kempe et al. 2003). In both cases, the core idea is that spreading happens in a physical or virtual network, when pairs of nodes are coming into contact. Traditionally, spreading was investigated under a static condition. However, with the rise of more fine-grained tracking systems, which are able to log timestamped contact information, there is a growing body of research studying the spreading problem based on temporal networks (e.g., Bajardi et al. 2012; Valdano et al. 2015).

The optimal selection of monitoring nodes for outbreak detection is a challenging task. Leskovec et al. (2007) address this problem in one of the most influential research works in the field. The authors propose a near-optimal outbreak detection strategy that uses greedy optimization for selecting a (small) set of nodes to be monitored. Crucially, the nodes are selected once and are then monitored constantly. Leskovec et al. propose three optimization goals: detection likelihood (DL), time until detection (DT), and the population that is affected by an outbreak (PA). The approach works well when the network is static and the edges do not vary over time (e.g., a water distribution network). However, for temporal networks, an optimal set of nodes selected based on a past period may not generalize well to a future period of time, especially if the network topology changes rapidly (Bajardi et al. 2012; Leskovec et al. 2007). In general, we may expect central nodes to be suitable for effective monitoring strategies, as they tend to become infected before others (Christakis and Fowler 2010).

In our previous article (Sterchi et al. 2020), we attempt to maximize the likelihood of detecting outbreaks of a disease at a given time *t*. We define an outbreak of a disease as a spreading process that is initialized by one particular node in the network becoming infectious. This seed node then transmits the disease to at least one connected node in the network. As long as a node is infectious it can further transmit the disease upon contact with another node. In Sterchi et al. (2020), we slightly modify Leskovec et al.’s approach by optimizing the set of monitoring nodes on a given day *t* with respect to a large number of simulated outbreaks over the period $[t-b, t]$ with $b=30, 60, 90$ days. More concretely, on a given day *t*, we select the nodes that detect the most outbreaks, or, in other words, nodes that are most often reached by outbreaks. The sets of optimal nodes may vary depending on *t*. As a consequence, we were restricted to the first of the objectives proposed in Leskovec et al. (2007), namely the optimization of the detection likelihood. In the present article, we avoid this restriction by replicating the approach proposed by Leskovec et al. By applying their method to two empirical timestamped contact networks and comparing the results with simple heuristic strategies that select nodes based on their centrality, we provide further empirical evidence for the greedy optimization method in the context of outbreak detection.

The approach consists of two main steps: (1) we simulate a large set of possible outbreak scenarios according to a simple *susceptible-infected-recovered* (SIR) propagation model (Barrat et al. 2008). The simulations are run on a past period where contact data are available. (2) we select nodes using a greedy strategy such that the three objectives mentioned above (DL, DT, and PA) are optimized on this past period. The size of the monitoring set, *k*, depends on the monitoring resources available. Importantly, we assume that the cost of monitoring a node is the same for all nodes.

The present study has two main objectives. First, we aim to investigate the characteristics of the set of nodes selected according to the greedy optimization approach proposed by Leskovec et al. (2007), as compared to simpler heuristics for node selection (based on centrality). Second, we want to analyze the generalization property, i.e., how well is a set of nodes optimized on a past period suited for outbreak detection on future data. Equivalently, we could ask how strongly the temporal-topological structure of a network changes over time leading to strong variations in the set of optimal nodes over time. These questions have also been partially covered by Leskovec et al. (2007) and subsequent studies. However, we believe that an application of the approach in Leskovec et al. (2007) to networks that are relevant for disease spreading among humans or livestock holdings is still lacking. The reader should bear in mind that this paper does not aim to examine a fully realistic disease spreading scenario. Instead, we use a simple SIR model which makes this paper comparable to many other works in the field of optimal surveillance and control (Bajardi et al. 2012; Holme 2018; Colman et al. 2019; Schirdewahn et al. 2017). However, as will become clear later on, the greedy approach is model-agnostic and can be applied to any spreading model that can be simulated on networks.

## Related work

Greedy optimization of submodular set functions goes back to the seminal work by Nemhauser et al. who provide “worst case bounds on the quality of the [greedy] approximations.” Nemhauser et al. (1978). More concretely, they show that the solution found by greedily optimizing a submodular set function will be no worse than $1-1/e$ times the often intractable optimal solution ($\approx 63\%$). This result had a profound effect on research communities in many different fields, as it provided a theoretical foundation for applying the simple greedy optimization algorithm to set functions. Unsurprisingly, many papers followed in the footsteps of Nemhauser et al. (1978). A popular application of greedy optimization for submodular set functions arises in the context of influence propagation in (online) social networks. Kempe et al. (2003) show that a greedily selected set of individuals, which maximizes the propagation of influence in a (static) social network, will reach more individuals than if the initial set of individuals is selected based on network centrality measures, such as degree centrality. Crucially, their approach employs extensive Monte Carlo simulations of a stochastic diffusion process (e.g., independent cascades (IC) or linear threshold (LT) models) in order to find the set of possible influence paths and their probabilities. It is obvious that this procedure does not scale to very large networks, which impedes the application of this approach to modern-day online social networks. Therefore, Chen et al. (2010) adopt a heuristic approach for the IC model that is based on local tree-like structures approximating the influence paths of a node. Avoiding the expensive simulations results in good scalability and, at the same time, the performance is close to that of Kempe et al. (2003). Another approach to avoid the Monte Carlo simulations has been proposed by Panagopoulos et al. (2019) who suggest using observed diffusion cascades. Instead of simulating the influence spread from every node, they simply use the observed diffusion cascades that maximize the marginal gain, which can be shown to be submodular. Finally, Budak et al. (2011) examine a variation of the influence propagation problem, namely the so-called influence limitation problem, which can be formulated as a submodular function maximization problem. The influence limitation problem has important applications, such as minimizing the impact of misinformation campaigns.

Optimal outbreak detection and influence maximization are two closely related problems. Maximizing influence corresponds to finding a set of seed nodes that maximize the size of the set of nodes to which the influence will propagate. In contrast, for optimal outbreak detection we aim to select a set of nodes that catches the maximum number of possible spreading cascades (DL objective). Leskovec et al. (2007) demonstrate how greedy optimization can be applied in the context of outbreak detection. In their seminal work, they propose three objective functions: detection likelihood, detection time, and the share of the population that is affected by the spreading process. All three objectives satisfy submodularity and can thus be greedily maximized with theoretical guarantees. One particular improvement of Leskovec et al. (2007) compared to previous approaches is the so called lazy-forward function evaluation. It is based on the idea that if the current marginal gain of a node *i* is larger than all other nodes’ marginal gains from the previous iteration, submodularity implies that *i* will also exhibit the largest marginal gain in the current iteration. This can dramatically reduce the number of function evaluations. However, Leskovec et al.’s approach has a limited applicability in temporal networks. For example, they show that the optimal node set selected based on observed blog network information cascades in the past does not generalize well to future outbreak detection. The reason for this is that blog networks are inherently dynamic and may change over time, such that the optimal node set will change substantially over time. One of the main objectives of this article is to assess this generalization problem in the context of epidemic spreading on a sexual contact network and an animal transport network. As mentioned above, the computationally expensive simulations may impair their use on large or very dense networks. However, there have been recent contributions in this area that substantially improve the efficiency of spreading simulations on temporal networks. For example, Vestergaard and Génois (2015) propose an extension of the Gillespie algorithm to temporal networks. Currently, the most efficient simulation approach seems to be an event-driven algorithm that is described in detail in Kiss et al. (2017), St-Onge et al. (2019), Holme (2020).

Both the outbreak detection and the influence maximization problem have been addressed with inexpensive heuristics, such as simply selecting the most central nodes. For example, Budak et al. (2011) note that a heuristic based on selecting nodes with high degree centrality is comparable in performance to the (greedy) optimization approach. Christakis and Fowler (2010) provide an intuitive explanation as to why central nodes can be good detectors of outbreaks: central nodes tend to be infected sooner, as they are topologically closer to the average node in a network. This has been confirmed by Holme (2018) who finds that degree, i.e., the number of distinct neighbors, (for static networks) and strength, i.e., the total number of contacts, (for temporal networks) are generally the best structural detectors of outbreaks for low transmission probabilities. A similar result comes from Sun et al. (2014) who find that in a large-scale city-wide outbreak detection scheme the number of contacts of an individual acts as the best detector in terms of early warnings about an outbreak. Colman et al. (2019) assert that a strategy based on selecting high-degree nodes for outbreak detection can be suboptimal if a (static) network is highly modular and exhibits a rather low degree heterogeneity because those central nodes may be topologically close. Therefore, they propose strategies that select high-degree nodes in different parts of the network (modules or spatial regions). Taken together, these studies indicate that selecting central nodes for monitoring may be overall the optimal structural heuristic for outbreak detection.

A different idea for outbreak detection has been proposed in Bajardi et al. (2012) and Schirdewahn et al. (2017) who suggest to monitor nodes that (1) are infected many times by *deterministic* spreading cascades from every possible seed node and (2) exhibit low uncertainty about the origin of the outbreak. However, this approach assumes that the starting time of the outbreak is known and spreading cascades are only simulated from this one starting time. Assuming that the specific contact patterns that underlie an emerging spreading process are not available, Valdano et al. (2015) propose to monitor so called loyal nodes, i.e., nodes that have been shown to repeat contact patterns over past periods. They show that loyal nodes are more likely to be reached by infections than disloyal nodes.

## Problem formulation

Let information about contacts between individuals be organized as a network $G = (V, E)$ where *V* denotes the set of nodes (individuals) and *E* the set of edges (contacts between individuals). The edges in the network are timestamped. Accordingly, an edge can be represented as a triple $(v_i, v_j, t)\in E$ with $v_i, v_j \in V$ and *t* a timestamp, indicating when the contact took place. The network can be directed or undirected. We assume that a single node introduced a disease into the network. The disease is then propagated along the edges in accordance with a propagation model, which is assumed to be known. Here, we use the classical *susceptible-infectious-recovered* (SIR) model: nodes get infected with probability $\beta$ if they are in contact with an infectious node and the time until recovery follows an exponential distribution (Barrat et al. 2008; Holme 2018). The aim of the work presented here is to identify an optimal set of nodes ${\mathcal {S}} \subseteq V$ such that some objective function is maximized (or minimized). We consider the same three objectives as in Leskovec et al. (2007): the detection likelihood (DL), the time until detection (DT), and the population affected (PA) (i.e., the size of the outbreak at the time of detection). Obviously, we aim to maximize the first and minimize the second and third objective. Once we find the optimal set of nodes, we can constantly monitor them. Since our monitoring resources may be constricted, we set a limit *k* on the number of nodes we can monitor. Thus, the optimization problem (for the DL objective) is the following:

1 $$\begin{aligned} \max _{{\mathcal {S}} \subseteq V} \; DL({\mathcal {S}}) \quad \text {subject to} \; |{\mathcal {S}}| \le k \end{aligned}$$

Similarly, we want to find the optimal node set ${\mathcal {S}}$ such that $DT({\mathcal {S}})$ and $PA({\mathcal {S}})$ are *minimized*. Note that all three objectives are set functions that take a set of nodes ${\mathcal {S}}$ as the input and return a real number. For example, for the PA objective, the set function would return the aggregate outbreak sizes (over all outbreak scenarios) at the time of detection by a node in ${\mathcal {S}}$.

## Outbreak detection

The outbreak detection approach used here is to a large extent identical with the one proposed in Leskovec et al. (2007). However, the key distinction between the information spreading example in Leskovec et al. (2007) and the disease outbreak examples in this work is that in the latter case no underlying spreading data are available. Hence, we need to simulate a set of outbreak scenarios. In this section, we first provide the details about the optimization objectives and then discuss the simulation approach we are going to use. Finally, we propose a fast implementation of the greedy optimization process.

### Objective functions

**Fig. 1:**
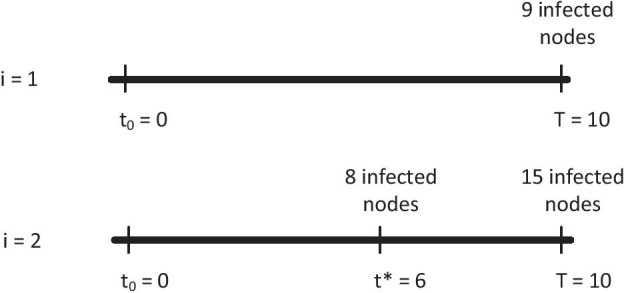
Two outbreak scenarios. In the first scenario ($i=1$), the outbreak is not detected by ${\mathcal {S}}$. The penalties for the DT, PA, and DL objectives are 10, 9, and 1, respectively. Obviously, the penalty reductions will be 0 for all three objectives. In the second scenario ($i=2$), the outbreak is detected at $t^*=6$ and the penalties are 6, 8, and 0. The corresponding penalty reductions are 4, 7, and 1

Leskovec et al. (2007) propose an alternative formulation of the optimization problem introduced in (1). Instead of directly optimizing the set functions, they suggest to maximize so called *penalty reductions*. The benefit of that is based on the fact that many nodes will have a penalty reduction of 0, which makes it a sparse optimization problem. The set of simulated outbreak scenarios can be denoted by ${\mathcal {I}}$ with *i* being one specific outbreak scenario. The penalty that scenario *i* incurs depends on the time it is detected by the monitoring set ${\mathcal {S}}$. We denote its detection time as $T(i,{\mathcal {S}})$ and the penalty for scenario *i* as $\pi _i(T(i,{\mathcal {S}}))$. For the DT objective, $\pi _i$ equals the time difference between the time of detection and the start of the outbreak in scenario *i*. Note that the penalty will assume some maximal value if a scenario is not detected. We can write this maximal penalty as $\pi _i(\infty )$. For the DT objective, the maximal penalty will be the time difference between the end of the simulation *T* and the start of the outbreak $t_0$. Similarly, the penalty for the PA objective corresponds to the size of the outbreak at the time of detection $T(i,{\mathcal {S}})=t^*$. For the PA objective, the maximal penalty corresponds to the size of the outbreak (number of infected nodes) at the end of the simulation. Finally, for the DL objective, the penalty is 0 for all outbreaks that are detected and 1 otherwise.With this in mind, we can define the penalty reduction for a given scenario *i* as $\pi _i(\infty ) - \pi _i(T(i,{\mathcal {S}}))$. For the DT objective, the penalty reduction corresponds to the difference between the end of the considered period and the actual detection time. For the PA objective, the penalty reduction corresponds to the difference between the final outbreak size at the end of the simulation and the outbreak size at the time of detection. Finally, for the DL objective, the penalty reduction will be 1 if ${\mathcal {S}}$ detects the outbreak in scenario *i*, and 0 otherwise. Figure 1 illustrates the computation of the penalty reductions with two simple examples.

The *weighted* penalty reduction (PR) for a given set of nodes ${\mathcal {S}}$ can then be written as follows:

2 $$\begin{aligned} PR({\mathcal {S}}) = \sum _{i \in {\mathcal {I}}} w_i \cdot (\pi _i(\infty ) - \pi _i(T(i,{\mathcal {S}}))) \end{aligned}$$

Note that in the remainder of this paper we will assume that all outbreak scenarios *i* have the same weight $w_i=c$ for some constant $c>0$ and therefore the weights have no influence on the optimal solution.

### Outbreak simulations

As mentioned before, we do not usually observe exact disease infection cascades. Hence, we need to simulate a large set $N=|{\mathcal {I}}|$ of possible spreading scenarios on which we will optimize the set of nodes to be monitored. For the simulations, we randomly select a seed node and the time the seed node starts infecting other nodes. We then use the SIR propagation model with known parameters to generate the spreading scenario. Simulating spreading scenarios can be expensive in terms of computation time, especially for networks with a large number of nodes or very dense networks (St-Onge et al. 2019). Kiss et al. (2017) propose an event-driven algorithm for static networks. The key idea is that, once a node is infected, we can directly sample i) all further transmission events originating from this node and ii) the node’s recovery time. By contrast, traditional simulation methods would require sampling for every edge between susceptible and infectious nodes and for all possible time steps after a node is infected. In general, this leads to a much larger number of random draws from the sampling distributions than for the event-driven approach. All transmission and recovery events are organized in a priority queue, which is ordered by the time of the events. The simulation stops when no more events are left in the priority queue. The event-driven algorithm has been extended to temporal networks by Holme (2020) whose implementation we use in this paper.

The simulation results are organized as an inverted index as suggested by Leskovec et al. (2007). For every node in the network, we store an identifier for each simulation run that infects it as well as the corresponding penalty reduction. The inverted index is ideal for fast lookups of penalty reductions by individual nodes.

### Greedy optimization

Optimizing the penalty reduction function in (2) is NP-hard as the number of possible sets ${\mathcal {S}}$ of a given size *k* becomes extremely large even for small graphs (Krause and Golovin 2014). As an example, consider a graph with 50 nodes. If we want to find a set of $k=10$ nodes that maximizes the expected penalty reduction, we would have to evaluate over 10 billion different sets. It is obvious that this problem becomes intractable very quickly. However, it can be shown that the penalty reduction function in (2) has some nice properties (Leskovec et al. 2007): it is 0 for empty node sets ($PR(\emptyset )=0$) and it is monotone and submodular. A set function *F* is monotone if for any two sets of nodes ${\mathcal {A}}$ and ${\mathcal {B}}$ with ${\mathcal {A}} \subseteq {\mathcal {B}} \subseteq V$, it holds that $F({\mathcal {A}}) \le F({\mathcal {B}})$. Furthermore, *F* is submodular if it satisfies

3 $$\begin{aligned} F({\mathcal {A}} \cup \{v\}) - F({\mathcal {A}}) \ge F({\mathcal {B}} \cup \{v\}) - F({\mathcal {B}})\;. \end{aligned}$$

for $v \in V \setminus {\mathcal {B}}$. Intuitively, submodularity implies diminishing marginal returns, i.e., the marginal contribution of a new node *v* is larger when added to a smaller set ${\mathcal {A}}$ compared to a larger set ${\mathcal {B}}$. A famous result by Nemhauser et al. (1978) states that non-negative monotone and submodular set functions can be greedily optimized and will yield a solution that is within $(1-1/e)$ of the optimal solution. In other words, the greedy solution of the penalty reduction functions will be guaranteed to be within 63% of the optimal (intractable) solution.

The greedy algorithm is simple and iteratively adds the node providing the largest marginal increase in the penalty reduction to the monitoring set and stops when the size of the set reaches *k*. In the most basic implementation, we would recompute all marginal gains at every step of the iteration, resort them and pick the node with the highest marginal gain. However, Leskovec et al. (2007) have shown that lazy forward evaluations (cf. Related work) allow us to *avoid* recomputing all marginal gains at every step, which can improve the running time significantly. We propose to use a binary heap data structure to facilitate the greedy optimization procedure and to avoid expensive resortings of marginal gains (Cormen et al. 2009). Algorithm 1 outlines our implementation of the greedy optimization algorithm with lazy forward evaluations for the DL objective. The algorithm can be easily adapted to the other objectives. Note that the functions $up\text{- }heap$ and $down\text{- }heap$ are standard utility functions of a max-heap implementation and the heap is sorted according to the marginal gains of nodes that are stored in an array $G[\;]$ of length |*V*|. We implemented the whole approach in C and the code is available on GitHub.^1^

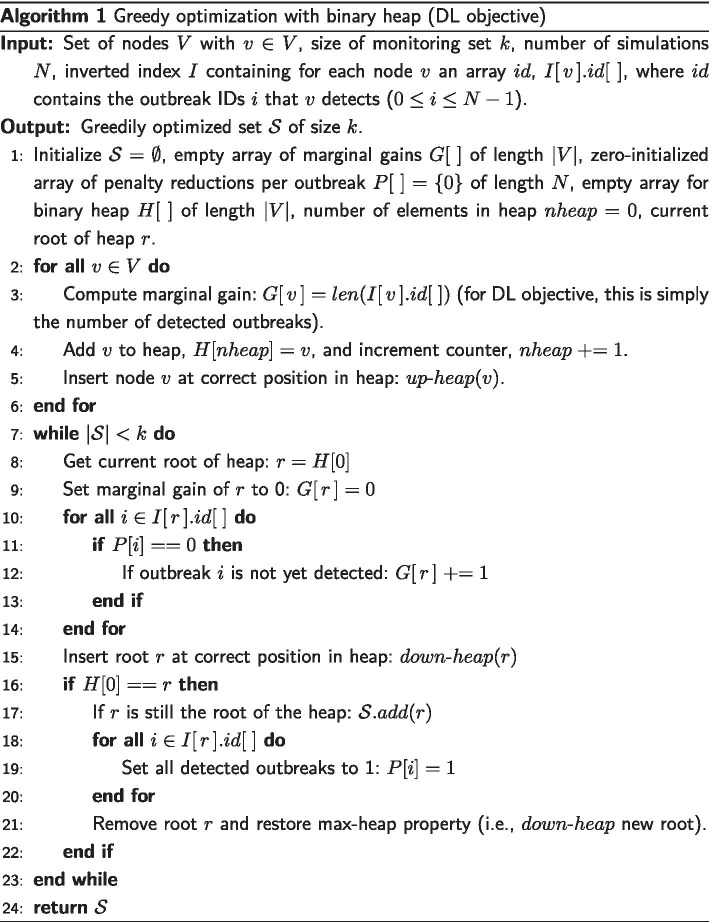



### Heuristic alternatives

In the sections below, where we present the results, we will compare the outbreak detection performance of the greedy optimization approach to simple heuristics where we select monitoring nodes based on structural properties. The following heuristics will be used:

#### Degree

Under certain conditions (cf. Related work), selecting high-degree nodes for monitoring seems to be a good strategy for efficient outbreak detection (Holme 2018; Colman et al. 2019). Note that we define the degree of a node as the number of distinct neighbors during some specified period of time. The degree is a popular centrality measure in the context of static networks, but its application is not limited to static networks and can be easily extended to temporal settings. For directed networks, we define the in-degree of a node *i* as the number of distinct neighbors with edges pointing to *i*. Similarly, we define the out-degree of a node *i* as the number of distinct neighbors node *i* points to.

#### Number of contacts

We can also count the total number of contacts of a node during a specified period of time. This is referred to as the *strength* of a node (Holme 2018). Nodes can be very different in terms of degree and number of contacts. For example, if a node is frequently in contact with one other node, then its degree will be 1 but its total number of contacts can be very high. Similarly to the definition of in- and out-degrees, we can distinguish in- and out-links for directed networks.

## Example 1: sexually transmitted infections

In the first example, we consider the spread of sexually transmitted infections (STIs). We use a dataset of timestamped sexual contacts between sex workers and their clients, which is widely used in research on temporal contact networks (Rocha et al. 2011; Valdano et al. 2015; Holme 2018; Antulov-Fantulin et al. 2015). The network is undirected and bipartite and contacts are reported on a daily basis. This rather low temporal resolution means that the order of contacts within a day is not known. We thus assume that consecutive transmissions must be strictly increasing in time. The full dataset covers a period of 6 years from September 2002 to October 2008. We will focus on the last 3 years of the dataset and consider *yearly* periods. That is to say, we will optimize node selection on a year’s worth of data. The 3-year network consists of 8220 sex-buyers and 5549 sex-sellers and thus 13, 769 nodes overall. A total of 40, 440 timestamped sexual contacts have been registered during this 3-year period (11, 189, 14, 399, and 14, 852, for each year respectively). The most active sex-seller is active 424 times during this 3-year period, while the most active sex-buyer totals 128 contacts in the same period.

For the simulation of the spreading scenarios, we use the SIR model with infection probability $\beta =0.3$ and an average recovery time of $\lambda ^{-1}=100$ days. These parameter values are in accordance with related work on STIs (Rocha et al. 2011; Antulov-Fantulin et al. 2015). Average run times of the simulations and the greedy optimization are given in Table 1.

**Table 1:** Average run time (and standard deviation) in seconds of the simulation of the outbreak scenarios and the greedy optimization for all three objectives and different minimal outbreak sizes

	Minimal outbreak size		
	1	2	5
*Example 1*			
Simulations	85.81s (6.24)	127.02s (4.40)	202.83s (6.55)
Greedy opt.	0.89s (0.02)	7.61s (0.28)	17.00s (0.08)
*Example 2*			
Simulations	17.79s (0.61)	21.57s (1.01)	161.18s (1.71)
Greedy opt.	0.04s (0.01)	0.09s (0.01)	0.18s (0.01)

Run times are averaged over 5 runs. For Example 1, we simulate 5 million scenarios and for Example 2, we simulate 1 million scenarios. All experiments were run on a Intel Core i7 machine (1.80 GHz) with 16 GB RAM

### Comparison of all optimization objectives

We greedily optimize the set of monitoring nodes for all three objectives on a year’s worth of data and call this the training phase. For this training phase, we simulate 5 million outbreak scenarios. As mentioned earlier, an outbreak is defined as a spreading process including at least one transmission of the disease between any pair of nodes. This is equivalent to a minimal outbreak size of 2. For the testing phase, we generate another 10, 000 outbreak scenarios (again with a minimal outbreak size of 2) either on the same period as for the training phase or on another period. In this subsection, we will train and test on the same period (year 3) and compare the relative performance of all three objectives. Results for other years are similar. We set $k=|V|$, that is, we optimize over the whole sequence of nodes in the network. Then, we sequentially compute the penalty reduction for the outbreak scenarios in the test set. The penalty reduction corresponding to the monitoring set of size $k=|V|$ (i.e., all nodes are monitored) is the maximal possible penalty reduction, i.e., *PR*(*V*). Hence, for any monitoring set ${\mathcal {S}}$ of size $k \le |V|$ we can compute a relative penalty reduction, *rPR*, as the fraction of the current penalty reduction and the maximal possible penalty reduction, that is,

4 $$\begin{aligned} rPR({\mathcal {S}}) = \frac{PR({\mathcal {S}})}{PR(V)}\;. \end{aligned}$$

**Fig. 2:**
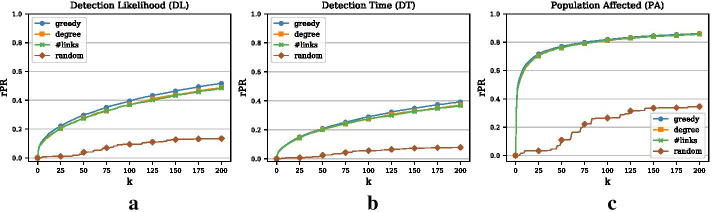
Relative penalty reductions (rPR) and comparison with benchmarks (Example 1). **a** Relative penalty reduction for DL objective. The x-axis represents the size of the monitoring set *k*. We only plot the curves for the top 200 nodes. **b** Relative penalty reduction for DT objective. (c) Relative penalty reduction for PA objective

Note that for the DL objective, the maximal possible penalty reduction is simply 10, 000 and the relative penalty reduction is thus equivalent to the fraction of detected scenarios. In Fig. 2 we plot the relative penalty reduction (rPR) for the greedy approach and the heuristic benchmarks. We plot the curves only for the top 200 nodes as this may correspond to the size of a realistic monitoring set that matches actual monitoring resources. As expected, the greedy curves for all three objectives are monotonically increasing but with diminishing marginal gains. For the DL objective (Panel (a)) we can see that the likelihood of detecting an outbreak with a monitoring set of 200 nodes is approximately 52%. What stands out is that for the PA objective (Panel (c)) we achieve a large penalty reduction very quickly with 75 nodes covering 80% of the maximal possible penalty reduction. Hence, the first few monitoring nodes for the PA objective seem to be part of the more densely connected part of the network where larger outbreaks happen and large penalty reductions are possible.

### Comparison with heuristic benchmarks

In Fig. 2, we also compare the greedy optimization approach to two simple benchmarks (as introduced above): (1) monitoring nodes with high degree and (2) monitoring nodes with the most contacts. In addition, we show results for a random baseline, where randomly chosen nodes are incrementally added to the monitoring set. The three Panels show the results for the DL, DT, and the PA objective, respectively. For the DL and the DT objective, the difference between the benchmarks and the greedy procedure is generally small but widens slightly with a larger monitoring set. For example, the detection likelihood of the degree heuristic for $k=200$ is roughly 49% as compared to the 52% of the greedy approach. In contrast, for the PA objective there is almost no difference between the greedy procedure and the benchmarks, which indicates that central nodes provide the largest penalty reductions for the PA objective.

**Fig. 3:**
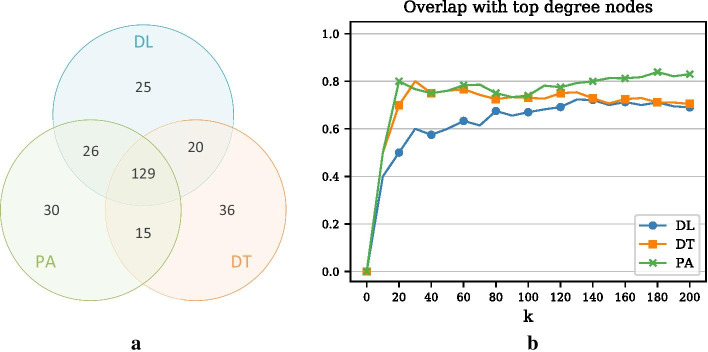
Comparison of three objectives and degree heuristic (Example 1). **a** Venn diagram representing the overlap of the top 200 nodes for the three different objectives. **b** Overlap of the three objectives with the degree heuristic (measured as the size of the intersection divided by *k*) for increasing *k*

Panel (a) of Fig. 3 shows the overlap of the node sets chosen according to the three objectives. Interestingly, the three optimal node sets share a total of 129 nodes (65%). Panel (b) plots the overlap of the three optimal node sets and the 200 most central nodes (degree heuristic) as a function of *k*. We can see that, especially in the beginning, the PA and the DT objective have a higher overlap with the degree heuristic than the DL objective. For $k=200$, the node set optimized with respect to PA contains 83% of the top degree nodes. In contrast, the overlap for the DL and DT objective is 69% and 71%, respectively. Overall, the PA objective chooses more high degree nodes than the other two objectives. A possible explanation for this may be that central nodes are located in the larger connected components where larger outbreaks and therefore larger penalty reductions are possible.

**Fig. 4:**
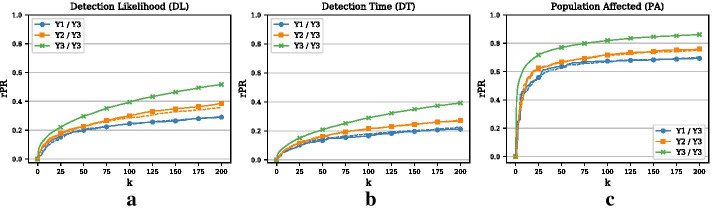
Training and testing on different periods (Example 1). **a** Relative penalty reduction for the DL objective and different training periods. Testing is always done in year 3 (Y3). For example, the blue curve indicates the detection performance when node selection was done based on the network of year 1 (Y1). The dashed curve represents detection performance of the degree heuristic. **b** Relative penalty reduction for the DT objective and different training periods. **c** Relative penalty reduction for the PA objective and different training periods

**Fig. 5:**
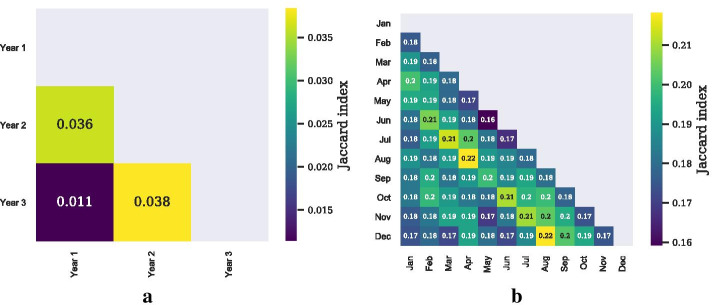
Overlap matrices (Example 1 and 2). **a** Overlap matrix between periods for Example 1. The overlap is measured by the Jaccard index. We compare the unique sets of static edges between periods. **b** Overlap matrix between periods for Example 2. Here, we compare the unique sets of static *directed* edges between periods

### Training and testing on different periods

All previous results use the same period for the training and the testing phase. However, in a realistic setting we will have to optimize the set of nodes on past data and hope that the monitoring set will also perform well on future data. We presume that the generalization to future data will be better if the network exhibits some regularities or some backbone that does not change much over time. Panels (a), (b), and (c) in Fig. 4 present the results for the DL, the DT, and the PA objective, respectively. As expected, training and testing on the same period (year 3) always outperforms training and testing on different periods. The figures also reveal that training on a period that is closer to the testing period (year 2 vs. year 1) is better in terms of penalty reduction. The dashed lines show the performance of selecting nodes by degree during the training phase. Panel (a) in Fig. 5 shows the overlap of edges between different (yearly) periods. For this analysis, we ignore the temporal information of edges and simply compare the static sets of edges $E_t$ with the set of (static) edges in previous years, e.g., $E_{t-1}$. The overlap is measured by the Jaccard index $J(E_t, E_{t-1}) = |E_t \cap E_{t-1}|\;/\;|E_t \cup E_{t-1}|$. From the figure, it can be seen that the overlap is larger for consecutive years, which accords with the results in Fig. 4. The overlap between year 1 and 3 is less than one third of the overlap between year 1 and 2 or year 2 and 3. Overall, however, the overlap between periods is small, which may be the result of the nature of this network that seems to exhibit only a small degree of organization.

**Fig. 6:**
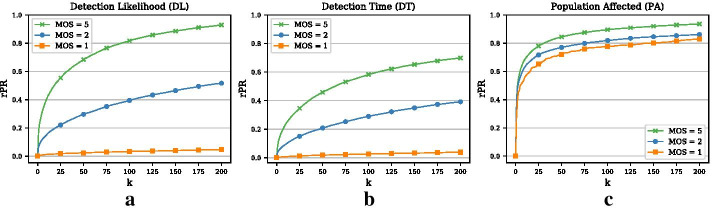
Effect of minimal outbreak size (Example 1). **a** Relative penalty reduction for the DL objective and different minimal outbreak sizes (MOS). **b** Relative penalty reduction for the DT objective and different MOS. **c** Relative penalty reduction for the PA objective and different MOS

### Minimal outbreak size

The results thus far are based on outbreak simulations that require at least one transmission of the disease. We can parameterize this by defining a minimal outbreak size (MOS). The higher the MOS, the more we bias the set of outbreak scenarios towards large outbreaks. For a minimal outbreak size of 1, the average outbreak size of the 10, 000 simulated outbreaks during the testing phase is 1.65 and the maximal outbreak affects 207 nodes. For a minimal outbreak size of 2 (this corresponds to the setting that we have considered so far), the average outbreak size is 10.75 and the maximal outbreak size is 313. Finally, for a minimal outbreak size of 5, the average outbreak size is 25.88 and the maximal outbreak size is 347. It is obvious that the performance of the outbreak detection methods is strongly affected by the way we simulate the outbreak scenarios. If we bias them towards larger outbreaks, then the detection performance will be better as large outbreaks occur in the more connected part of the network where the highly central nodes will catch many of them. This is illustrated in Panels (a)–(c) in Fig. 6. The effect of a larger MOS is more pronounced for the DL and the DT objective than for the PA objective. This is a direct consequence of the PA objective: the largest penalty reductions can be achieved for large outbreaks. Hence, even for a minimal outbreak size of 1, the greedy optimization procedure is going to focus on the nodes that catch the few large outbreaks early on.

## Example 2: animal disease spreading

In the second example, we consider reported pig movements in Switzerland during the year 2017 (Sterchi et al. 2019). The movements are reported as directed transports between two animal holdings and contain a timestamp that indicates the day of transport. All movements to slaughterhouses are discarded, as the goal is to monitor farms and catch outbreaks before the final transport to the slaughterhouse. One movement is not necessarily equivalent to one direct transport between the two holdings, but may be part of a tour where multiple different herds of pigs are transported together. Here, we restrict our focus to disease transmission through introduction of new animals in an animal holding and neglect possible transmissions between herds during the transport. In line with (Bajardi et al. 2012), within-farm dynamics are ignored and a movement is regarded as a directed contact potentially transmitting a disease from one farm to the other. We consider monthly networks because a period of 30 days has been considered especially important for animal movement networks (Dubé et al. 2008; Nöremark et al. 2011). This duration corresponds to the silent spread phase, which is typically the maximum time a disease can spread before incidental detection. The full dataset (1 year) contains 6, 664 nodes and 48, 980 directed edges. The monthly networks contain on average 4, 082 edges. The most active node is involved in 615 recorded movements over the course of the year.

As in Example 1, we use a SIR model to simulate the spreading scenarios. In accordance with related work (Bajardi et al. 2012; Valdano et al. 2015; Schirdewahn et al. 2017), we simplify the spreading process by assuming that the infection probability $\beta$ does not depend on the size of the herd that is transported. We use a rather high infection probability of $\beta =0.6$ that takes into account the nature of the contact, which is more prone to transmission (multiple possibly infected pigs get introduced into a susceptible farm) than for example in the case of human disease spreading. Note that (Bajardi et al. 2012; Valdano et al. 2015) use even higher infection probabilities (see their supplementary material). The average time until recovery we use is $\lambda ^{-1}=7$ days as in Bajardi et al. (2012). Note, however, that in Bajardi et al. (2012) the time until recovery is deterministically set to 7 days, whereas we use the stochastic version where the time until recovery stochastically varies around the average value of 7 days. Average run times of the simulations and the greedy optimization are given in Table 1.

**Fig. 7:**
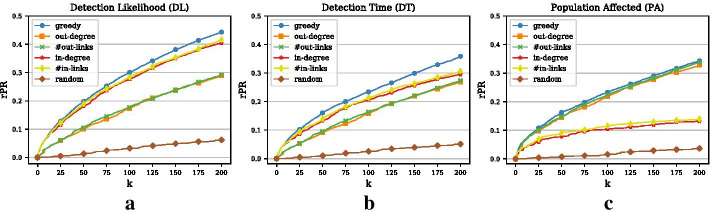
Relative penalty reductions (rPR) and comparison with benchmarks (Example 2). **a** Relative penalty reduction for DL objective. The x-axis represents the size of the monitoring set *k*. We only plot the curves for the top 200 nodes. Since the network is directed, we compare with both the in- and out-degree heuristic as well as the in- and out-links heuristic. **b** Relative penalty reduction for DT objective. **c** Relative penalty reduction for PA objective

### Comparison of all optimization objectives

As mentioned above, the relevant time period we study in this example is 1 month. All results below show the results for the month of December. Results for other months are similar. Since the network of animal movements is smaller than the network in Example 1 and we consider a shorter period of time (1 month vs. 1 year), we only simulate 1 million outbreak scenarios for the training phase. As before, we focus on a minimal outbreak size of 2, i.e., there must be at least one transmission of the pathogen from one animal holding to another. For the testing phase, we again simulate 10, 000 outbreak scenarios and we only present results for the top 200 nodes. Panels (a)–(c) in Fig. 7 show the greedy curves for the three objectives. In contrast to Example 1, the relative penalty reduction for the DL objective is higher than for the other two objectives. As we will show below, the outbreak sizes are considerably smaller and less skewed to the right in this Example as compared to Example 1. As a result, it is harder to optimize the PA objective since smaller outbreaks that are harder to detect contribute comparatively more to the overall penalty reduction. Based on the DL curve we can say that the overall likelihood of detecting an outbreak given a monitoring set of 200 nodes is about 44%.

### Comparison with heuristic benchmarks

If we compare the greedy optimization method with the heuristic benchmarks for the three objectives, we get an interesting result. For the DL and DT objective (Panel (a)–(b) in Fig. 7), the best heuristics select nodes based on their in-degree or the number of in-links. For the PA objective (Panel (c)), the opposite is the case: the best heuristic consists of selecting nodes with high out-degree or number of out-links. The reason for this is that high in-degree nodes (or nodes with many in-links) are in general more likely to catch outbreaks originating at different seed nodes, which crucially contributes to the DL objective. By contrast, monitoring nodes with a large out-degree (or many out-links) will lead to large penalty reductions for the PA objective since outbreaks with a large spreading potential are caught at the beginning of the spread. Even though this result seems somewhat trivial, it is important and shows that, especially in the case of directed networks, different objectives lead to different sets of optimal nodes.

**Fig. 8:**
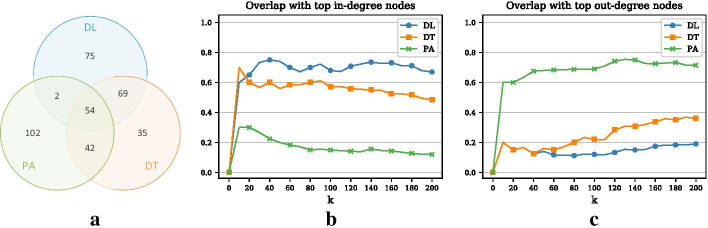
Comparison of the three objectives and in- and out-degree heuristics (Example 2). **a** Venn diagram respresenting the overlap of the top 200 nodes for the three different objectives. **b** Overlap of the three objectives with the in-degree heuristic (measured as the size of the intersection divided by *k*) for increasing *k*. **c** Overlap of the three objectives with the out-degree heuristic for increasing *k*

Panel (a)–(c) in Fig. 8 confirm the finding from the previous paragraph. The overlap of nodes between the three objectives (54 nodes) is far smaller than in Example 1 and more than half of all nodes selected by the PA objective (102 nodes) are not selected by the other objectives (Panel (a)). We can also see that the DL and to a lesser degree the DT objective share nodes with the in-degree heuristic (Panel (b)) whereas the PA objective shares a lot of nodes with the out-degree heuristic (Panel (c)).

**Fig. 9:**
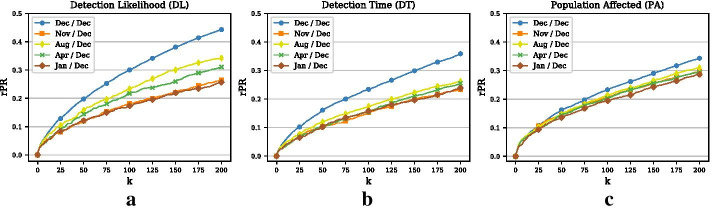
Training and testing on different periods (Example 2). **a** Relative penalty reduction for the DL objective and different training periods. Testing is always done in December. **b** Relative penalty reduction for the DT objective and different training periods. **c** Relative penalty reduction for the PA objective and different training periods

### Training and testing on different periods

Since we use monthly networks in this example, we use 12 networks (January–December) for the training phase and test the results on outbreaks simulated on the December network. Panels (a)–(c) in Fig. 9 show the results for the DL, the DT, and the PA objective, respectively (for visual clarity, we only plot the interesting curves). What is striking is that using the August or the April network seems to be better, especially for the DL objective, than using the November network, which would be closer to the testing phase in terms of time. This indicates that there are some sort of 4-month seasonal patterns in the networks, a result that has been found in our previous work as well (Sterchi et al. 2020). A possible explanation for this somewhat surprising result may be the production cycle that is inherent to the Swiss pig industry and which may be related to the length of the gestation period of pigs ($\sim 4$ months).

Panel (b) in Fig. 5 shows the overlap matrix for Example 2. From the matrix, it can be seen that the overlap is almost consistently highest between month *t* and month $t-3$. For example, the Jaccard index assumes its highest value (0.22) between December and August and between August and April. This is in accord with our observations above. Overall, we can say that the overlap between periods is generally larger compared to the sexual contact network in Example 1, which may be due to the higher degree of organization and coordination in this system.

### Minimal outbreak size

We have noted above that the way we simulate the outbreak scenarios can strongly impact the performance of the different outbreak detection objectives. Here, we also use different minimal outbreak sizes (MOS). If $MOS=1$, the average outbreak size for the evaluation data (10, 000 outbreaks) is 1.06 while the maximal outbreak for this setting affects 7 nodes. If we require at least one transmission (minimal outbreak size of 2), then the average outbreak size increases to 2.25 with a maximal outbreak size of 14. For a minimal outbreak size of 5 nodes, the average outbreak size is 6.03 and the largest outbreak affects 21 nodes. The outbreak sizes are considerably smaller than in Example 1, which has multiple reasons. First, we only consider periods of 1 month as compared to yearly periods. Second, the spreading process is strongly limited by the short average recovery time of $\lambda ^{-1}=7$ days. Finally, the directed nature of the network restricts disease transmissions more strongly than the undirected network in Example 1.

**Fig. 10:**
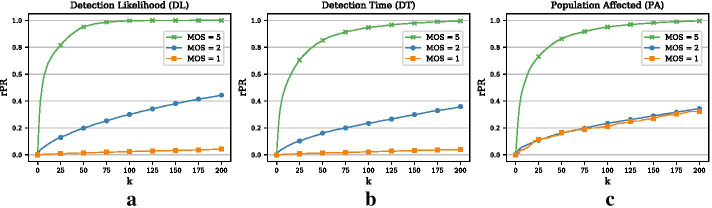
Effect of minimal outbreak size (Example 2). **a** Relative penalty reduction for the DL objective and different minimal outbreak sizes (MOS). **b** Relative penalty reduction for the DT objective and different MOS. **c** Relative penalty reduction for the PA objective and different MOS

Panels (a)–(c) in Fig. 10 show the performance of the greedy approach for the DL, the DT, and the PA objective, respectively. In all three graphs, the performance is considerably better if we require outbreaks of 5 or more nodes. In that case, it is enough to monitor 100 nodes in order to catch all of the 10, 000 outbreaks (DL objective). For the PA objective, we observe that the performance of minimal outbreak sizes of 1 and 2 are very similar and thus the larger outbreaks with large penalty reductions must be very similar for the two configurations.

## Discussion and conclusion

The aim of this paper is to extend the greedy optimization method for outbreak detection (Leskovec et al. 2007) to the setting of disease outbreak detection on temporal contact networks. We focus on the same three objectives as in Leskovec et al. (2007): maximizing the detection likelihood, minimizing the time until detection, and minimizing the population affected by an outbreak. These three objectives can be shown to be submodular. Finding the optimal set of nodes that maximizes or minimizes these objectives would be NP-hard. However, due to the submodularity property we have theoretical guarantees that the solution found by greedy optimization is a good approximation to the theoretical optimum.

We used two temporal contact networks to evaluate the greedy approach and to compare it to heuristic benchmarks based on the centrality of a node. One dataset contains sexual contacts and thus represents a possible scenario for the spread of STIs. The other dataset represents directed contacts between Swiss pig farms and may be relevant for the spread of infectious diseases among animals, such as the foot-and-mouth disease (FMD) or African swine fever (ASF). In line with the related work (Rocha et al. 2011; Bajardi et al. 2012; Holme 2018; Colman et al. 2019; Schirdewahn et al. 2017; Antulov-Fantulin et al. 2015), we use a SIR model, which, in most cases, corresponds to a strong simplification of the true processes at work. Note however, that our approach is not constrained by the assumption of a SIR model and it is straightforward to adapt it to any propagation model that can be simulated efficiently.

The results in this paper show that selecting nodes for surveillance based on the greedy optimization approach performs at least as well as the heuristic methods and sometimes even slightly better. However, heuristics such as selecting high-degree nodes are computationally much cheaper and therefore provide a valuable alternative to the optimization-based approach. This corroborates earlier findings in influence maximization (Budak et al. 2011). Moreover, the degree heuristic has been shown to perform well by Holme (2018) for small outbreaks and by Colman et al. (2019) for static networks with small modularity and high degree heterogeneity. This brings us to another important implication of the greedy approach. It may outperform degree-based heuristics by a larger margin for highly modular networks, especially for the DL objective. However, more work is required to establish the connection between the network structure and the optimization-based selection of monitoring nodes. Another important aspect noted by Holme (2018) is that the degree of a node (or the number of links) is a local measure and estimating it does not require knowledge of the network. The fact that the heuristics are comparable in performance to the greedy optimization approach is promising and it can thus be suggested that efficient outbreak detection is possible with limited computational resources and does not require knowledge of the full network structure.

An interesting aspect of our results is that the type of nodes chosen for surveillance depends strongly on the type of network we consider. For the undirected network of sexual contacts, the node sets selected according to the three objectives overlap strongly and high-degree nodes seem to be crucial for all objectives. On the other hand, we observe very dissimilar node sets for the directed network of pig transports. Nodes that satisfy the PA objective (large out-degree) are different from nodes satisfying the DL and DT objective (large in-degree). This may have to do with the structure of the Swiss livestock industry, which is organized in a hierarchical and decentralized way where farms specialize on a specific task in the production process (Sterchi et al. 2019). As a result, farms at the beginning of the production process tend to have a large out-degree and are different from farms at the end of the production process typically exhibiting a large in-degree.

Another important point is that optimizing node selection on past time periods and applying the set of selected nodes for outbreak detection on future time periods may not work well for networks that have strong structural variations between periods. For example, the sexual contact network has a very small overlap in edges between periods that may be due to a lack of organization in this system. Valdano et al. (2015) describe this as a “lack of an intrinsic cycle of activity characterizing the system.” On the other hand, the animal movement network exhibits larger overlaps between periods, indicating a more organized system. While for the sexual contact network, selecting nodes based on a period that is close to the testing period works best, the animal movement network exhibits seasonalities that suggest selecting nodes in period $t-3$ for monitoring in *t*. More research should be undertaken to investigate whether or not we can further optimize node selection on past data by optimizing on different past periods and possibly aggregating results over different time periods.

A further implication of this study is that the creation of the set of (realistic) outbreak scenarios is not as straightforward as it seems. We introduced a minimal outbreak size in order to set a lower bound for the size of an outbreak. However, this results in a heavily skewed distribution of outbreak sizes as a large portion of the outbreak scenarios will exhibit the minimal outbreak size. By increasing the minimal outbreak size we can force the simulation approach to generate larger outbreaks, which will improve the detection performance as demonstrated in this paper. This implies that the detection performance of the greedy approach and the heuristics is strongly affected by the way we simulate outbreaks (mostly in terms of size). Future work on defining and creating *representative* sets of realistic outbreak scenarios is needed.

Taken together, the findings of this study suggest that the optimal nodes for outbreak detection are often the highly central nodes in the network because they are either infected early on or they are the origin of a potentially large outbreak themselves. Furthermore, the question which past period should be used to select nodes for future outbreak detection depends on the degree of organization a system exhibits but also on seasonal patterns. The findings of our paper may be somewhat limited by the fact that we only consider two example networks and one specific spreading process, namely a SIR model. To develop a full picture of the greedy optimization approach for outbreak detection and its comparison to heuristics, additional studies are needed that examine its performance over a range of parameter values and for different network structures and spreading models.

## Data Availability

The pig movement data contain private information and cannot be shared publicly. For research purposes, a data request can be sent to Identitas AG, Stauffacherstrasse 130A, 3014 Bern, Switzerland. The escort data are openly available and can be downloaded from https://journals.plos.org/ploscompbiol/article?id=10.1371/journal.pcbi.1001109. The code for this study is available on GitHub: https://github.com/martinSter/epi-outbreak-detection.

## Abbreviations

ASF: African swine fever
DL: Detection likelihood
DT: Detection time
FMD: Foot-and-mouth-disease
IC: Independent cascades
LT: Linear threshold
MOS: Minimal outbreak size
PA: Population affected
PR: Penalty reduction
rPR: Relative penalty reduction
SIR: Susceptible-infected-recovered
STI: Sexually transmitted infection

## Footnotes

1 https://github.com/martinSter/epi-outbreak-detection.
